# Reports on Hospitals of the United Kingdom

**Published:** 1913-12-13

**Authors:** Henry Burdett


### REPORTS ON
### Hospitals of the United Kingdom.

By SIR HENRY BURDETT, K.C.B., K.C.V.O.

SERIES III.

### GREAT YARMOUTH HOSPITAL.

The present hospital was built in 1887, but it might have been built thirty years earlier, for its notable faults of construction ought never to have been found in a hospital only twenty-five years old. The hospital is well lighted and airy, but its plan is one to make angels weep. It is not too much to say that the expert will find here striking lessons of how not to plan a hospital. There is one long corridor running the whole length of the frontage. At one end of this corridor is a ward of twelve beds or so, and at the other end a second ward of twelve beds, so that the nursing of these two wards, which is in charge of one sister, is rendered as difficult and costly as possible. There is no direct communication between several wards and their lavatories and bathrooms. To enter these it is necessary to go out of the ward into the main corridor and to pass some distance down it, and then to proceed along a passage, at the side and end of which are the conveniences in question. Anything more inconvenient or objectionable for many reasons it would be difficult to devise.

### Reconstruction and Equipment Necessary.

It is not necessary to go into details of the fittings and furniture, which may be dismissed with the remark that, though some of them have been renewed and are fairly good, the ward kitchens especially are mean in appearance, and could be and ought to be smartened up. There is an absence of cupboards, housemaids' closets, and other necessaries which would enable the whole of the ward unit to be kept neat and smart. As it is at present everything has to be put wherever a vacant space can be found, and there is in consequence an appearance of untidiness very often, which we fear is unavoidable in the circumstances. Again, the bathrooms and lavatories are lumbered up with all sorts of things for which suitable accommodation ought to be provided elsewhere. The plan of the first floor is bad, with the same defects, though in a portion of the building some of these defects have been somewhat modified from the worst.

Comparatively recently a children's ward has been added, which is approached from the backyard of the hospital up two long flights of steps. It possessed no means of communication with the main building until recently, when an approach was made through the lavatories attached to one of the women's wards. This serious omission and the faults which the plan of the children's ward exhibit, though it is infinitely better than earlier portions of the hospital, prove to demonstration the importance of hospital committees not sanctioning the plans of additional buildings unless they have had them examined and reported upon by some competent person, with practical knowledge of the requirements of a modern hospital and the working arrangements that should be provided for in all such plans. From what we have said it will be apparent to the initiated that the task of administering and managing this hospital is necessarily costly and difficult. Besides, the plan is so inconvenient as it stands that it materially increases the labour of the staff and necessarily discourages efficiency. It is objectionable, too, on moral grounds, because the absence of the necessary accommodation and the consequent crowding of the lavatories and bathrooms must have the effect of discouraging adequate ablutions on the part of the patients.

### Good Work in the Wards.

We were particularly anxious to ascertain the quality of the work which was being done in the wards by the staff. We were very pleased to make the acquaintance of the house surgeon, Mr. James C. Watt, of the Glasgow University and Western Infirmary, who had come to Yarmouth to do practical work, and has evidently done it with infinite credit to himself and advantage to the hospital. It reflects great credit on the staff, as Mr. Watt was able to demonstrate in the wards and by the results recorded in the hospital books, that this hospital now undertakes every kind of case, however difficult, and that the old practice of sending severe cases elsewhere is discontinued altogether. The results of the treatment are excellent, and the Yarmouth people are fortunate in having this convincing evidence of the excellent quality of the medical and surgical facilities they have within their reach. The operation theatre is well equipped, but resembles that at Lowestoft in the sense that much of the floor space is occupied by trolleys and other furniture, the reason being, it was explained, that everything has to be laid out for operations owing to the smallness of the nursing staff. Each hospital necessarily does its work in its own way, but the difficulties here presented by the furniture and appliances seem to us so serious that we very much regret not having had an opportunity of being present at an operation to see exactly how the surgeons are able to overcome the various obstructions, which appeared to be so unnecessarily numerous.

### The Committee's Point of View.

The committee of this hospital appear to have kept before them the commendable object of making both ends meet exactly, year by year. This they have tried to do without realising the enormous increase in the cost of medical, and especially of surgical, treatment in a hospital where the staff, as at Yarmouth, endeavour to bring about the highest state of efficiency. It seems, as far as we could gather, not to be realised by the committee at Yarmouth that, where they have a progressive and efficient medical staff, that staff deserves and must have the support of a progressive and up-to-date committee. The developments in treatment during the last twenty-five years, and especially during the last ten years, necessarily make the old economical policy, which invariably seems to have dominated the committee at Yarmouth, improper and out of date. It is perfectly clear from a study of the accounts and a close examination of the medical and surgical work since 1910 that it is the imperative duty and privilege of the committee to take the necessary steps to increase at once the income of this hospital by at least £1,000 per annum. During the last few years there has been an average deficiency of nearly £500, which has been unavoidable, and was indeed meritorious, because it represented a justifiable outlay in return for which the people of Yarmouth and district have materially benefited, directly by the restoration to health of many patients afflicted with grievous illness, and indirectly by giving to the better-class residents the protection and security of having amongst them the type of medical practitioners who are capable of rendering the highest service in the most difficult cases.

### A Supreme and United Effort Called For.

The income in the past, owing to the relatively small non-progressive voluntary revenue obtained, is so small as to be unworthy of the inhabitants of Yarmouth. These Norfolk folk, when judged by other communities of equal size, do not as a whole contribute anything like the sum which they can properly give and ought to give to the hospital. Their total gifts to the hospital during the year ended May 1913, including annual subscriptions and donations, with the sums raised through the Hospital Sunday and Saturday Funds, amounted to only £2,518! The accounts exhibit no evidence of any special efforts made to increase the income by enlisting the services of the majority of the people who are well able to give, for other places have proved the ability and willingness of these classes to give in small sums a considerable total each year. Take, for instance, the women. There is no organisation amongst the women of Yarmouth, and we would strongly urge the committee, through the Mayoress or some other lady of influence, to start and organise a Ladies' Association, which could readily, we are confident, increase the income by £500 a year. There appears to be no branch of the League of Mercy working in the Yarmouth district or in the county, so that the field is perfectly open for the ladies to organise a system of small gifts, to divide Yarmouth into districts or wards, to place in charge of those wards active and well-known residents who will obtain the co-operation of a number of ladies of every class of the community, and to work under captains of streets, who in this way should be able to enlist small contributors of one shilling a year in monthly subscriptions of one penny and upwards, which subscriptions should be called for every month. We are confident, from experience in many other towns, that the women of Yarmouth are capable, and we have confidence that they will be willing, if the committee will show enough energy to commence the movement, to bring into the hospital exchequer some hundreds of pounds each year.

The committee should also interest the fisher women and girls who spend two months each year in Yarmouth in the herring season. These women, we are advised, number about 10,000, and are paid on the average for the season something like £350,000 in wages—that is to say, the average fisher woman's earnings are £35 per head for the season. If they on an average are working ten weeks in Yarmouth, and will each contribute one penny per week, as we believe they would gladly do, the sum they might contribute annually should be at least £400 a year. In this connection we may point out that at the time of our visit there were several fisher girls who were patients, and the fisher girls as a class use this hospital and benefit considerably from it. There are also the fishermen and the working-classes generally, who at present appear to contribute only £173 per annum. That sum, we are confident, might be increased if an organisation of energetic workers can be recruited who will bring the claims of the hospital to the notice of the working-men and get their leaders and foremen interested in the work. Then, too, there are the large number of residents in Yarmouth, which is a very prosperous place, especially during the season, when the younger inhabitants might usefully organise special efforts to interest the visitors and to bring in as annual subscribers the large number of householders who do not at present contribute anything directly to the hospital. The point that we wish to make for the committee's consideration is that the basis from which they raise the present income is too narrow; that it requires broadening in some directions such as we have indicated. The way to get money is not only to provide efficient medical and general management, but to avoid constant appeals for small sums by one great organised effort to awaken the whole population to the needs of the Yarmouth Hospital, and so to bring up the revenue of this institution to a sum which, on an average, will provide an adequate income to cover each year's necessary expenditure. The present committee seems to us to lack driving force and faith. Yarmouth now has some excellent men of business, and we would appeal directly and personally to each one of them to lend a hand to this hospital and to supply business energy and direction to the work of permanently raising the revenue from £3,500 to £4,000 per annum. This they can readily do if they will only exercise a little self-denial by putting their shoulders to the wheel in the present emergency, and so lift the hospital out of the rut, financially speaking, into which it has fallen.

What the Hospital Wants.

This hospital needs some things which the committee ought to provide without delay. Amongst them are: A better and fuller equipment in the wards and in the various ward kitchens and ward adjuncts. Another thing which should be provided forthwith is a housemaids' closet for every ward, so that the untidy makeshift of drying clothes and other things in the bathrooms and elsewhere within the ward unit could be forbidden. It needs an adequate number of cupboards for the reception of linen and other stores, so that the work of the staff may be rendered less difficult and the general smartness and efficiency of the hospital be everywhere improved. The lavatories are at present full of all sorts of things which ought never to find a place there. Indeed, as we have said, some lavatories were so crowded up with things as to make it difficult if not impossible for the patients to use the washing basins with any comfort. The wards lack adequate and hygienic lockers, the present ones being unsightly and largely useless. There should also be a number of chairs provided in the wards, so that the patients and their friends may have somewhere to sit. In a word, the equipment of this hospital at present is starved, a defect which is particularly noticeable in the older portions of the hospital. We commend these facts to the urgent attention of a few Yarmouth men and women who may be able to afford, and would be much better for affording, if they will allow us to say so, the means adequately to equip one ward at least. Such spontaneous gifts would record their high intelligence and demonstrate the depth of human sympathy which animates them in regard to the sick.

**Lowestoft and Norfolk and Norwich Hospitals will be reported on next week.**

